# The Impact of COVID-19 on Healthcare Worker Wellness: A Scoping Review

**DOI:** 10.5811/westjem.2020.7.48684

**Published:** 2020-08-17

**Authors:** Jacob Shreffler, Jessica Petrey, Martin Huecker

**Affiliations:** * University of Louisville, Department of Emergency Medicine, Louisville, Kentucky; † University of Louisville, Kornhauser Library, Louisville, Kentucky

## Abstract

At the heart of the unparalleled crisis of COVID-19, healthcare workers (HCWs) face several challenges treating patients with COVID-19: reducing the spread of infection; developing suitable short-term strategies; and formulating long-term plans. The psychological burden and overall wellness of HCWs has received heightened awareness in news and research publications. The purpose of this study was to provide a review on current publications measuring the effects of COVID-19 on wellness of healthcare providers to inform interventional strategies. Between April 6–May 17, 2020, we conducted systematic searches using combinations of these keywords and synonyms in conjunction with the controlled vocabulary of the database: “physician,” “wellness, “wellbeing,” “stress,” “burnout,” “COVID-19,” and “SARS-CoV-2.” We excluded articles without original data, research studies regarding the wellness of non-healthcare occupations or the general public exclusively, other outbreaks, or wellness as an epidemic. A total of 37 studies were included in this review. The review of literature revealed consistent reports of stress, anxiety, and depressive symptoms in HCWs as a result of COVID-19. We describe published data on HCW distress and burnout but urge future research on strategies to enhance HCW well-being.

## INTRODUCTION

The COVID-19 pandemic has resulted in significant burdens globally. Detrimental effects include high rates of infection and death, financial hardships faced by individuals, stress related to known and particularly unknown information, and fear of the uncertainty regarding continued impact. Healthcare workers (HCWs), at the heart of the unparalleled crisis of COVID-19, face challenges treating patients with COVID-19: reducing the spread of infection; developing suitable short-term strategies; and formulating long-term plans. HCWs must also continue to successfully treat non-COVID patients and maintain personal responsibilities, including taking care of their families and themselves. The psychological burden and overall wellness of HCWs has received heightened awareness, with research continuing to show high rates of burnout, psychological stress, and suicide.^1^

HCWs experience emotional exhaustion, which may lead to medical errors, lack of empathy in treating patients, lower productivity, and higher turnover rates.^2^ The ability of HCWs to adequately cope with stressors is important for their patients, their families, and themselves. Providers vary in levels of psychological resilience, the ability to positively adapt to adversity to protect themselves from stress.^3^ Prior to COVID-19, wide-ranging research had established the multifactorial nature of stressors in healthcare: electronic health record duties; insurance and billing issues; any patient dissatisfaction; and balancing busy work-life schedules.^4^

HCWs must continue to balance these existing obstacles to wellness while facing the unique challenges of a pandemic. Literature from severe acute respiratory syndrome and Middle East respiratory syndrome can provide insight on the stress, trauma, psychological morbidities, and successful interventions, but the body of evidence for the impact COVID-19 on HCW wellness is evolving alongside the pathogen. The purpose of this study was to provide a review of current publications measuring the effects of COVID-19 on wellness of the HCWs to inform interventional strategies.

## METHODS

Between April 6–May 17, 2020, we conducted systematic searches in PubMed, Embase, Cochrane, Clinical Key, and Web of Science using combinations of these keywords and synonyms in conjunction with the controlled vocabulary of the database: “physician,” “wellness,” “wellbeing,” “stress,” “burnout,” “COVID-19,” and “SARS-CoV-2.” Results were filtered to English-language publications, retrieving a total of 107 references. We examined references in included papers and relevant excluded papers for additional studies, and a non-systematic search in Google Scholar was conducted as well. After those selections were added and duplicates were removed, 185 distinct references remained for screening.

To reduce risk of bias, titles and abstracts were screened for eligibility by two independent reviewers, with a third available in the event of disagreements. Papers presenting original data regarding the evaluation or management of physicians’ well-being during the COVID-19 pandemic were included for full analysis. Some publications indexed as correspondence did contain data, so article type was not an automatic exclusion criterion, nor was study design and quality of methodology. While papers on infection control practices, personal protective equipment (PPE), or wellness in other types of HCWs were not actively sought out, any retrieved by the strategies were retained. We excluded articles without original data, research studies regarding the wellness of non-healthcare occupations or the general public exclusively, other outbreaks, or wellness as an epidemic (Figure).

## RESULTS

We included 37 studies in this review. Multiple themes emerged from the current literature on how COVID-19 has impacted HCW wellness. The majority of studies focused on the psychological impact of COVID-19, including stress and anxiety measurements. Some evaluated burnout and sleep quality. A small portion of the studies used qualitative methodology. We have provided a summary of the articles below.

### Stress, Fear, Anxiety, Depression

In light of the many known and unknown effects of COVID-19, exploration of stress, fear, anxiety and symptoms of depression were prevalent in the included studies, with many focusing on frontline HCWs.

#### Frontline Workers

Researchers assessed anxiety levels in 512 frontline healthcare workers in China, finding a prevalence of 12.5%.^5^ The authors found HCWs who had direct contact with COVID-19 patients were at higher risk for anxiety.^5^ Frontline workers were also a focus by Lu et al in 2299 HCWs (2042 medical staff and 257 administrative staff). The authors found that medical staff had greater fear, anxiety, and depression levels than administrative staff. Additionally, the investigators found that HCWs working on frontlines in departments more impacted by COVID-19 (ie, emergency department, intensive care unit, infections disease) were at greater risk for anxiety and depression and psychological disorder.^6^

A total of 5062 HCWs were surveyed by Zhu et al to measure psychological impact of COVID-19.^7^ The authors measured stress, depression, and anxiety, discovering that 29.8% of respondents reported stress, 24.1% reported anxiety, and 13.5% reported depression. Women, individuals with history of mental disorders, and HCWs with infected family members were more vulnerable to undesirable health consequences of stress, anxiety, and depression.^7^

Liu et al measured distress, anxiety, and symptoms of depression in 4679 Chinese HCWs.^8^ Results showed the prevalence of anxiety and distress was about 16% each; 34.6% of respondents experienced depressive symptoms. The investigators discovered that risk factors for developing the mental health concerns aforementioned included living alone, being a nurse, being on the frontline, and middle age.^8^

Li et al measured the vicarious traumatization phenomenon in three groups: frontline nurses, non-frontline nurses, and general population. Frontline nurses had lower levels of trauma than both the general public and non-frontline nurses. The authors hypothesized that frontline nurses have better training to deal with crisis.^9^ Similar findings were discovered in 470 HCWs in Singapore.^10^ Results showed non-medical workers had greater anxiety and stress compared to medical workers. Among the 470 HCWs, 14.5% experienced anxiety with 7.7% experiencing levels of concern for post-traumatic stress disorder.^10^

Liang et al compared HCWs in COVID-19 associated departments to other HCWs. They found a significant portion of HCWs experienced clinically depressive symptoms, but no significant differences between frontline HCWs and non-frontline HCWs.^11^ A study by Cai et al measured the psychological impact of COVID-19 on 534 frontline medical-staff members.^12^ The authors found that HCWs experienced anxiety about their own and their family’s safety (along with their patients) but maintained the professional obligation to effectively complete their work. The authors found that older staff had increased stress related to (lack of) PPE and longer work hours. Coping strategies used by the HCWs included adhering to strict protective measures, following isolation guidelines, and exhibiting a positive mindset.^13^

Guo et al studied 11,118 HCWs in China. Results showed that risk factors for anxiety and depression were being younger, employed as a nurse, and being a frontline HCW. Within the sample, about 5% experienced middle to high levels of anxiety and about 13.5% experience middle to high depression levels.^13^ Lai et al found that female gender predicted greater risk of psychological stress in a study that examined depression, anxiety, insomnia, and distress in 1257 HCWs.^14^ HCWs experienced high incidence of depression (50.4%), anxiety (44.6%), and insomnia (34%). The majority of HCWs reported distress (71.5%), with women, nurses, frontline workers, and those working in Wuhan, China, having higher negative health outcomes.^14^

Dai et al discovered that geographic location was a risk factor for distress in 4357 HCWs. The results showed that 39.1% of HCWs experienced distress; living in Wuhan, being isolated, worrying about family members and working on the frontline were risk factors for experiencing distress.^15^ HCWs were chiefly concerned about infection in colleagues or family members.^15^ Geographic location was also a risk factor in a study in Italy comparing stress and anxiety in healthcare workers (n = 167) to the general population (n = 186). Likelihood of exposure to disease (HCWs and individuals in highly affected Northern Italy) predicted increased stress and anxiety. Overall, HCWs reported higher levels of worry compared to the general population.^16^

In a letter to the editor, Du et al reported smartphone survey data on frontline HCWs in Wuhan. HCWs from two hospitals and one outreach team answered multiple questions during a five-day period. The outreach team members appeared more prepared psychologically, had more supplies, and had improved sleep, stress, and levels of depression compared to frontline workers. Fifty-nine percent of respondents had moderate to severe perceived stress, with 12.7% having at least mild depressive symptoms and 20.1% having anxiety. Those at greatest risk were HCWs who felt less prepared, had less family support, felt less self-efficacy, perceived a higher level of stress, and those with poor sleep quality. Fear of self and colleague infection represented a top source of stress.^17^

#### Unspecified/Other Healthcare Workers

Access to PPE was a key focus by Zhang et al, who surveyed 304 HCWs in Iran. The authors found that 28% of HCWs experienced anxiety, 20.1% experienced distress, and 30.6% experienced depression. Furthermore, the study revealed that access to PPE resulted in both improved physical health and job gratification and ultimately led to less distress among HCWs.^18^ Delgado et al measured HCW personal safety perception in 936 workers in Latin America. Overall, HCWs lacked sufficient PPE and felt limited support from human resources and public officials.^19^

Preparedness to fight COVID-19 was examined in 158 HCWs in England. The authors found that HCWs desired more actions (including proper education) to feel confident to fight COVID-19, particularly in the collection and management of samples.^20^ Suleiman et al conducted a similar study on preparedness for the COVID-19 outbreak, surveying 308 physicians in Jordan. Individuals with protocols in place and accessible PPE reported higher levels of readiness. Furthermore, 90.9% of respondents reported feelings of anxiousness regarding the transmission of the disease and fear of the increase of the volume of infected patients. The large majority (96.4%) of HCWs were worried about transmitting COVID-19 to loved ones.^21^

Chew et al measured stress and anxiety in HCWs in 906 HCWs in Singapore and India. The results showed that 48 (5.3%) HCWs faced moderate to very severe depression and 79 (8.7%) had moderate to extremely severe depression. Additionally, 54 (6%) HCWs experienced moderate to extremely severe stress or moderate to severe distress. After correcting for confounders, the authors noted an association between incidence of prior month physical symptoms and emotional distress during COVID-19.^22^

In Wuhan, China, Kang et al measured mental health and psychological wellbeing using a survey in 994 HCWs. The study revealed that 28.6% of the sample had moderate to severe mental disturbances, with young women affected the most. Within the study population, subjects who accessed mental health amenities had improved relationship between exposure risks and mental health.^23^

Jiang et al measured psychological impact by comparing self-efficacy and loneliness of 205 HCWs in Hubei, China. Medical staff with lower self-efficacy had higher likelihood of loneliness. The authors noted that individuals experiencing loneliness may choose undesirable coping tactics (eg, substance use).^24^ The protective effect of a committed relationship surfaced in 194 physicians surveyed in Oman. The researchers revealed that individuals who were married and older experienced less stress compared to other HCWs. Additionally, the authors found that females may be more susceptible to stress.^25^

Some physician-specific studies occurred in the included literature. Chen et al surveyed pediatricians on outcomes of stress and anxiety. Of 105 respondents, 90.5% of the sample were female and 18.1% reported working in high-risk areas. The authors noted particularly high self-reported depression and anxiety during the COVID-19 outbreak.^26^ In a study completed by Xu et al, the researchers surveyed 60 surgical staff during a period of COVID-19 outbreak and compared them with a separate group of 60 surgical staff in a non-outbreak period in China. The results showed that HCWs surveyed during the outbreak period had significantly higher levels of anxiety and depression.^27^ One researcher used the Beck Anxiety Inventory to measure anxiety in multiple sclerosis fellows in Iran. The authors had 14 respondents and only two individuals had mild levels of anxiety.^28^

A focused look at dentists and dental hygienists assessed the COVID-19 impact in Israel. Among the 338 surveyed, individuals with previous illness and those worried about infection from patients were inclined to higher levels of distress. HCWs in committed relationships and those with superior levels of self-efficacy reported less stress.^29^

### Burnout

Wu et al specifically measured burnout in 220 oncology medical staff working in Wuhan, China. Using the validated and widely deployed Maslach Burnout Inventory-Medical Personnel (MBI), they compared levels of burnout in frontline and other HCW groups. Frontline HCWs had significantly lower levels of burnout and were less worried about becoming ill compared to those in the “usual ward” group. The authors noted two possible explanations: frontline HCWs may perceive more control over their situation and may appreciate a closer proximity to decision-makers (with more timely provided information) compared with the other HCWs.^30^

Cao et al used the MBI to measure burnout and emotional distress in 37 HCWs. They found that the levels of burnout and emotional distress were not highly elevated within their sample. Connecting with family members via technology or telephone was the most prevalent coping mechanism. The study showed that 29.7% of the sample had issues obtaining proper sleep.^31^

### Sleep

Some researchers focused specifically on COVID-19’s impact on HCW sleep. Xiao et al surveyed 180 medical staff members on social support, anxiety, stress, self-efficacy, and sleep quality to determine the effects of COVID-19.^32^ The authors found that social support correlated significantly with both self-efficacy and quality of sleep. Anxiety and stress were significantly associated, leading to negative impacts on both self-efficacy and sleep. The authors recommended HCWs to take advantage of support systems, including family and friends to stabilize emotions, share experiences, and maintain social connections, thus reducing anxiety intensities and enabling quality sleep.^32^

Huang and Zhao measured sleep, anxiety, and depressive symptoms in 2250 HCWs. The authors compared HCWs’ results to individuals from the general population. Results showed that HCWs were more likely to experience poor quality sleep and develop psychological issues.^33^ Qi et al also measured sleep in their survey of 1306 (801 frontline) HCWs in China. The authors found that frontline HCWs had advanced anxiety, depression, and prevalence of sleep disturbances compared to non-frontline HCWs. Furthermore, the authors found that female frontline HCWs had higher prevalence of sleep disturbances compared with male frontline HCWs.^34^

Zhang W et al found that medical HCWs had higher levels of insomnia, anxiety, depression, somatization, and obsessive-compulsive symptoms compared to non-medical HCWs in 2182 respondents in China. Risk factors for worsened mental health included living in a rural area, being female, and having contact with infected COVID-19 patients.^35^ Finally, insomnia was measured in HCWs in China involved in the COVID-19 outbreak. Of the 1563 respondents, 36.1% reported insomnia symptoms. Insomnia risk factors included lower levels of education, working in a unit with isolation, being a physician, lack of support, having high levels of uncertainty, and being worried about infection. The authors called for interventions for insomnia for HCWs.^36^

### Qualitative Approach

Some researchers used qualitative methods to gain better insight into the impact of COVID-19 on HCW wellness. Liu et al interviewed nine nurses and four physicians in Hubei. Respondents described many challenges of COVID-19 including fear of infection, exhaustion, and working in a new context. Despite these challenges, the HCWs felt that they were fully responsible to care for their patients as it was part of their duty, demonstrating an immense vow to their profession. The authors noted that workplace safety including access to PPE was a top priority.^37^

Sun et al interviewed 20 nurses who provided care for COVID-19 patients in China. The study results indicated that anxiety and fear were prevalent in the early stages of the outbreak, leading to feelings of helplessness. The authors noted some healthy coping strategies, including team encouragement and rational thinking. Furthermore, the authors found that reflection and developing a sense of professional responsibility resulted in growth. Finally, the researchers discovered that the nurses experienced both negative and positive emotions concurrently.^38^

Sethi et al also used a qualitative approach to develop open-ended questions for 290 HCWs in Pakistan. They found that HCWs were anxious, overworked, and felt financially unstable. Furthermore, HCWs reported challenges in taking care of both their professional lives and their households.^39^

### Healthcare Workers Who Became Ill

This review did not intend to describe exposure, infection rate, or mortality of healthcare providers during the COVID-19 pandemic. Two publications that appeared in our literature search described providers who became infected with the virus, but we refer readers to reviews focused on this topic.^40^,^41^ Ran et al examined risk factors for HCWs who developed acute respiratory infection in Wuhan China. The authors found that longer hours, higher risk clinical setting, and suboptimal hand cleanliness were risk factors for infection.^42^

Researchers in Wuhan, China, studied 103 HCWs who had become infected with COVID-19. These HCWs answered questions on perceived cause of infection and psychological changes. Results showed that the large majority (84.5%) of HCWs who became infected felt it was due to their work setting, with nurses’ top three perceived causes being suction care, swab collection, and other nursing requirements. Physicians perceived highest risk in physical examination and tracheal or manual ventilations. During the isolation period, 88.3% of these HCWs experienced stress or emotional deviations. The study showed that persons who were experiencing distress were apprehensive about their own health in addition to transmitting it to loved ones.^43^

## DISCUSSION

The review of literature revealed consistent reports of stress, anxiety, and depressive symptoms in HCWs as a result of COVID-19. Multiple studies confirmed significant anxiety regarding patient care in addition to the possibility of infecting their families.^12^,^16^,^30^ Access to appropriate PPE remains of paramount importance to help physicians feel physically safe. With sufficient PPE, individuals feel more protected from infection, which may lessen fear of infecting loved ones. Women and individuals in high-risk areas may have more negative psychological health outcomes. Furthermore, both individuals on the frontline and other HCWs are susceptible to distress and negative health outcomes including anxiety, poor quality sleep, and feelings of isolation. Interestingly, some frontline workers experienced better mental health outcomes. The sense of vocation / purpose in work, along with greater control of environment, could explain these findings.

Given the relative novelty of this crisis, no published studies have collected data on interventions to improve psychological health and overall wellness for HCWs who face COVID-19 specific challenges. Suggestions to alleviate the burden on HCW mental health have been provided by researchers both for COVID-19 and in other crises. We found no studies that measured the same sample before and after COVID-19 to determine how wellness or stress changed within the same individuals. Researchers could compare previous datasets on provider wellness to measure and quantify effects of COVID-19.

Generally recognized for their emotional resilience, HCWs must now face additional layers of responsibilities and mental and physical hardships.^1^ We remain uncertain about the timeline and actions needed to effectively combat this virus but hope to reduce severity of current and future waves of infection.^44^ Targeted individual and organizational strategies for mental health and overall wellness for HCWs are critical for these courageous individuals. Based on the narrative review of the literature, we believe the following are necessary strategies for HCW wellness provided in the Table.

## LIMITATIONS

The research on effects of COVID-19, and physician wellness in general, continues to rapidly evolve.

Therefore, updated reviews will be necessary in the coming months. The present review was limited by search strategies designed to retrieve publications with a focus on overall well-being, burnout, or stress; thus, studies exclusively about the physical protection, infection, and transmission rates within this population may not have been retrieved. Future research should consider assessing the psychological burden placed upon HCWs by practical and physiological aspects of disease. Finally, there was limited literature from US providers due to this scoping review being conducted in the earlier stages of the pandemic.

## CONCLUSION

We recognize the obstacles to implementing strategies to improve HCW wellness: financial barriers; worker engagement; etc.^2^ Burnout, stress, and the emotional burden of caring for sick patients were already affecting HCWs before COVID-19. Long-term effects of the worldwide pandemic remain unknown. We described published data on HCW distress and burnout but urge future research on strategies to enhance HCW wellbeing. To continue to provide uninterrupted, quality care, the healthcare workforce – human beings – must be empowered and encouraged to take care of themselves.^4^

## Supplementary Information



## Figures and Tables

**Figure:**
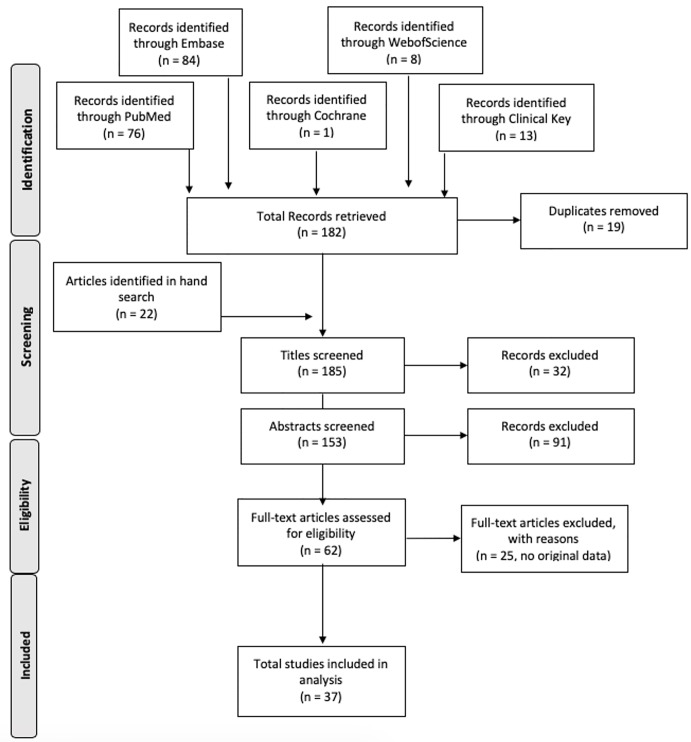
Process of systematic searches using combinations of “physician,” “wellness,” “wellbeing,” “stress,” “burnout,” “COVID-19,” and “SARS-CoV-2” to provide a scoping review on publications measuring the effects of novel coronavirus 2019 on wellness of healthcare workers.

**Table:** Strategies for healthcare worker wellness.

Immediate and individualized access to mental health resources. Short-term and long-term individualized wellness and mental health interventions to address the physical and emotional tolls of COVID-19. Individual AND organizational strategies to optimize wellness for healthcare providers in areas of nutrition, exercise, mindfulness, sleep quality, and reducing burnout. Quality, accessible PPE for all HCWs to provide security and reduce likelihood of infection for themselves and their loved ones. Opportunities to research and implement telehealth in a variety of settings to limit exposure to infection. Reduce stigma on mental health symptoms and the psychological impact of significant stressful events within HCWs. Development of new HCW community groups and encouragement of participation to allow connections and reduce feelings of isolation.

*PPE*, personal protective equipment; *HCW*, healthcare worker.
