# Supplementary material for: The Impact of COVID-19 on Healthcare Worker Wellness: A Scoping Review
Source: West J Emerg Med. 2020 Aug 17;21(5):1059–66. doi: 10.5811/westjem.2020.7.48684 (PMC7514392; doi:10.5811/westjem.2020.7.48684)
Supplement: Supplementary file 1 [file wjem-21-1059-s001.docx]

| Authors | Article Type | Study design | Sample Size | Population | Peer Review |
| --- | --- | --- | --- | --- | --- |
| Badahdah et al | Letter to the Editor | Survey | 194 | Physicians | Editorial |
| Cai et al | Clinical Research | Questionnaire | 534 | Frontline medical staff | Full |
| Cao et al | Letter to the Editor | Interviews | 37 | Medical workers | Editorial |
| Chen et al | Letter to the Editor | Questionnaire | 105 | Pediatric Medical Staff | Editorial |
| Chew et al | Article in Press | Questionnaire | 906 | HCWs | Full |
| Dai et al | Pre-print | Survey | 4357 | HCW | No |
| Delgado et al | Article | Survey | 936 | HCWs | Full |
| Du et al | Letter, Pre-proof | Survey | 310 | HCWs | Editorial |
| Guo et al | Original Research | Survey | 11118 | Medical staff | No |
| Huang & Zhao | Pre-proof | Survey | 7236 | General public, included 2250 HCWs | Editorial |
| Jiang et al | Preprint | Questionnaire | 205 | HCWs | No |
| Jin et al | Research | Questionnaire | 105 | HCWs | Full |
| Kang et al | Article in Press | Questionnaire | 994 | Doctors 183 and nurses 811 | Full |
| Lai et al | Original Research | Survey | 1257 | HCWs | Full |
| Li et al | Article in Press | Questionnaire | 740 | General public 214, Frontline nurses 234. nonFL nurses 292 | Full |
| Liang et al | Letter | SDS/SAS scores | 59 | Doctors and nurses in COVID and non COVID departments | Editorial |
| Liu C et al | PrePrint | Questionnaire | 512 | Medical staff | No |
| Liu Q et al | Original Research | Interviews | 13 | Nurses (9) and Physicians (4) | Full |
| Liu Z et al | Pre-print | Survey | 4679 | Doctors and nurses | No |
| Lu et al | Article | Survey | 2299 | Medical (2042) and administrative (257) hospital staff | Full |
| Moghadasi | Letter to the Editor | Questionnaire | 14 | MS fellows | Editorial |
| Prescott et al | Pre-proof | Questionnaire | 158 | Frontline HCWs | Editorial |
| Qi et al | Pre-print | Questionnaire | 1306 | Frontline 801 and non-frontline 505 medical workers | No |
| Ran et al | Research | Retrospective Questionnaire | 72 | HCWs | Editorial |
| Sethi et al | Original Article | Survey | 290 | HCWs | Full |
| Shacham et al | Article | Survey | 338 | Dentists and dental hygienists | Full |
| Simione et al | Original Research | Questionnaire | 353 | HCWs and students 176 | No |
| Suleiman et al | Article | Questionnaire | 308 | Doctors | Full |
| Sun et al | Major Article | Interviews | 20 | Nurses | Full |
| Tan et al | Letter: Brief Research Report | Questionnaire | 470 | Medical (296) and nonmedical (174) HCW | Editorial |
| Wu et al | PreProof | Survey | 220 | Medical staff 1:1 COVID: nonCOVID wards | Editorial |
| Xiao et al | Clinical Research | Questionnaire | 180 | Doctors and nurses in respiratory medicine, fever clinics, or ICU | Full |
| Xu et al | Letter, Pre-proof | Questionnaire | 120 | Surgical medical staff | Editorial |
| Zhang C et al | Original Research | Questionnaire | 1563 | Medical staff | Full |
| Zhang S et al | Letter | Mixed Survey and Interview | 304 | HCWs for surveys, Interviewed (5) Doctors and nurses | Editorial |
| Zhang W et al | Clinical Note | Survey | 2182 | Medical health workers (927) and Non-medical health workers (1255) | Full |
| Zhu et al | PrePrint | Questionnaire | 5062 | HCW | No |
